# Fucoidan from Marine Macroalgae: Biological Actions and Applications in Regenerative Medicine, Drug Delivery Systems and Food Industry

**DOI:** 10.3390/bioengineering9090472

**Published:** 2022-09-14

**Authors:** Grace Sathyanesan Anisha, Savitha Padmakumari, Anil Kumar Patel, Ashok Pandey, Reeta Rani Singhania

**Affiliations:** 1 Post-Graduate and Research Department of Zoology, Government College for Women, Thiruvananthapuram 695014, India; 2 Department of Marine Environmental Engineering, National Kaohsiung University of Science and Technology, Kaohsiung City 81157, Taiwan; 3 Institute of Aquatic Science and Technology, National Kaohsiung University of Science and Technology, Kaohsiung City 81157, Taiwan; 4 Center for Energy and Environmental Sustainability, Lucknow 226029, India; 5 Centre for Innovation and Translational Research, CSIR-Indian Institute of Toxicology Research, Lucknow 226001, India; 6 Sustainability Cluster, School of Engineering, University of Petroleum and Energy Studies, Dehradun 248007, India

**Keywords:** fucoidan, antimicrobial, antiviral, anticancer, antioxidant, regenerative medicine, food packaging

## Abstract

The marine macroalgae produce a collection of bioactive polysaccharides, of which the sulfated heteropolysaccharide fucoidan produced by brown algae of the class Phaeophyceae has received worldwide attention because of its particular biological actions that confer nutritional and health benefits to humans and animals. The biological actions of fucoidan are determined by their structure and chemical composition, which are largely influenced by the geographical location, harvest season, extraction process, etc. This review discusses the structure, chemical composition and physicochemical properties of fucoidan. The biological action of fucoidan and its applications for human health, tissue engineering, regenerative medicine and drug delivery are also addressed. The industrial scenario and prospects of research depicted would give an insight into developing fucoidan as a commercially viable and sustainable bioactive material in the nutritional and pharmacological sectors.

## 1. Introduction

The marine macroalgae produce an arsenal of economically important polysaccharides with promising applications that form the basis for an escalating blue economy. They have potential pharmaceutical, nutraceutical and cosmetic benefits which make them applicable as functional ingredients for human health. Among the different algal polysaccharides, sulfated polysaccharides, such as fucoidan, have grabbed the attention of researchers. They have different biological actions, such as anticoagulant, antithrombotic, antidiabetic, anti-obesogenic, immunomodulation, anticancer and antiproliferative activities [1,2,3]. 

According to the International Union of Pure and Applied Chemistry (IUPAC) nomenclature system, fucoidan specifically designates the heterogeneous marine sulfated polysaccharides which are copiously found in the cell wall matrix of various species of brown algae [4]. The cell walls of brown algae consist of an amorphous matrix of acid polysaccharides, such as fucoidan and alginic acid, which are linked to each other by proteins, giving structural integrity and flexibility to the seaweed [5]. Generally, fucoidan constitutes about 5–10% of the dry algal biomass, which varies based on the species and the seasons.

The algal polysaccharides demonstrate a wide range of structural diversity, and their functionality is largely concomitant with their structural divergence [6]. The algal polysaccharides are hydrolyzed by several carbohydrate-active enzymes which help in their utilization via different metabolic pathways [7]. Recently, the pharmacological properties of secondary metabolites and other bioactive substances in marine macroalgae have been reviewed by Priyanka et al. [8], emphasizing their antimicrobial, antitumor, anti-inflammatory, antidiabetic, antiprotozoal, antiviral, and antioxidant activities. Zayed et al. [9] reviewed the biogenic sources and outstanding bioactivities of galactofucans/G-fucoidans, giving insights into the possibilities of novel drug discoveries. The current review focuses on the structure, chemical composition, extraction and purification of fucoidan from the marine brown macroalgae. The review also highlights the supreme biological actions and health benefits of fucoidan, such as antioxidant, anticancer, hepatoprotective and neuroprotective actions, with special reference to their application in regenerative medicine, drug delivery systems and the food industry.

## 2. Marine Macroalgal Sources of Fucoidan

Macroalgae, also called seaweeds, are taxonomically categorized into the phyla Chlorophyta (green algae), Rhodophyta (red algae) and Phaeophyta (brown algae). Brown seaweeds are benthic, and they inhabit the coastal ecosystems in temperate and cold-water seas [10]. Brown macroalgae produce the fucoidan polysaccharide [4] (Table 1). The brown seaweeds, such as *Ecklonia cava*, *Ascophyllum nodosum*, *Cladosiphon okamuranus*, *Undaria pinnatifida*, *Saccharina longicruris*, *Saccharina latissima*, *Sargassum polycystum*, *Laminaria japonica*, *Fucus vesiculosus* and *Fucus serratus*, are abundant sources of fucoidans [11,12,13,14,15,16,17].

## 3. Cultivation of Marine Macroalga

Among the cultivated macroalgae, brown macroalgae and red macroalgae are more predominant than green macroalgae. Brown, red and green macroalgae contributed, respectively, 47.3%, 52.6% and 0.05% of the total global production in 2019. The predominantly cultivated brown macroalgal species are *Laminaria/Saccharina japonica* (Japanese kelp or kombu) and *U. pinnatifida* (Japanese wakame) [24,25].

A transition from wild stock harvesting to aquaculture can ensure the sustainable supply of seaweed biomass and meet the increasing demand for various applications in the industrial and health sectors. Various aquaculture methods are in practice for the offshore and onshore cultivation of marine macroalgae. 

Onshore cultivation of macroalgae is land-based cultivation conducted in closed systems, such as tanks, raceways, ponds or lagoons, wherein seaweeds are exposed to sunlight and suspended in nutrient-supplemented water by agitation. In Germany, for example, macroalgae are grown in artificial seawater in land-based systems [24]. Land-based cultivation has the advantage of monitoring and real-time adjustments of cultivation conditions and being unaffected by tides, waves and wind. On the contrary, the main limitations of land-based cultivation are the need for extensive land and the high cost of infrastructure building, workforce and energy for the maintenance of farm conditions [26]. Another major obstacle in the large-scale onshore cultivation of macroalgae is the need for a feasible nutrient source [27]. The wastewater effluents from municipal sources or biogas plants can be used as nutrient-rich media for the cultivation of macroalgae. Sebök and Hanelt [27] reported that processed biogas digestate with rich nutrients can be used for the cultivation of marine macroalgae, increasing the algal biomass. 

Offshore cultivation of macroalgae is conducted in seawater at a certain distance from the coastline. Kelp growth, raft cultivation, floating cultivation, fixed off the bottom, long lines and rock-based farming are the offshore practices for macroalgae cultivation. Kelp farming is performed by vertical and horizontal rope systems. Raft farming makes use of long line single rafts and grid raft blocks. In cultivation, using ropes and nets, the seaweed seedlings can be either attached directly to the ropes or grown via transplantation. In the transplantation technique, the seedlings are first grown in indoor greenhouse tanks, following which the small fronds are transplanted onto the ropes and nets in the sea. Cultivation using ropes and nets is cheap and easy to maintain and hence they are the most prevalent techniques of macroalgae farming [28]. In rock-based farming, algae are directly planted on the ocean bottom or attached to an artificial substrate. Though offshore cultivation of macroalgae is promising and cost-effective, the harsh environmental conditions of the sea are a major threat that necessitates measures to mitigate the environmental risks [26]. 

Currently, integrated multi-trophic aquaculture (IMTA), with macroalgae playing a prime role, is emerging as an environmentally friendly farming practice [29]. IMTA comprises the farming of two or more aquatic species from different trophic levels and has the advantage of high production efficiency with reduced waste generation and improved bioremediation services for the ecosystem. In IMTA, complex interactions occur between the algae and its microbiome, wherein the bacteria release algal growth- and morphogenesis-promoting factors that promote the large-scale production of macroalgae [30].

## 4. Composition, Structure and Physicochemical Properties of Fucoidan

**Figure 1:**
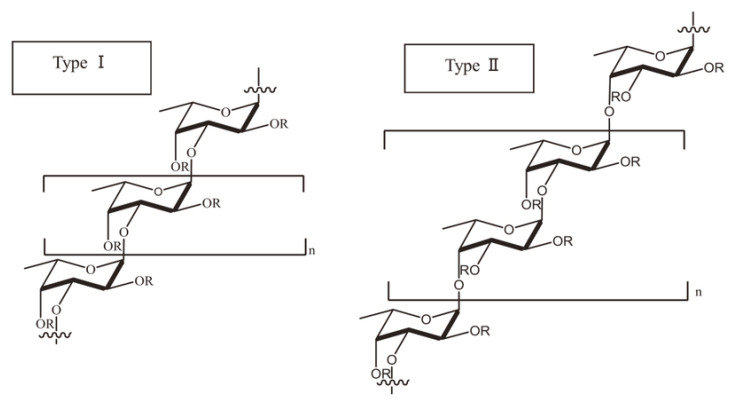
Structure of fucoidan. Type I fucoidan has repeating units of α-(1→3)-linked α-L-fucopyranose and Type II fucoidan has alternately repeating units of α-(1→3)- and α-(1→4)-linked α-L-fucopyranose [31].

The fundamental subunit of fucoidan is fucose (C_6_H_12_O_5_) which is a deoxyhexose sugar [3]. The amount of L-fucose in fucoidan outweighs other monosaccharides and accounts for more than 90% of its total sugar composition [4]. The other monosaccharides in fucoidan comprise varying proportions of uronic acid, galactose, glucose, xylose, mannose, rhamnose, arabinose and acetyl groups [17,31,32,33]. The composition and complexity of fucoidan polysaccharides vary among different species of brown macroalgae (Table 1) and are largely influenced by geographical location and seasonal variations [33,34]. The diverse chemical compositions are reminiscent of their differences in biosynthesis. Although fucoidans do not have a universal chemical structure [4,5], the scientific literature available to date suggests principally two structural types of fucoidans: Type I fucoidan has repeating units of α-(1→3)-linked α-L-fucopyranose and Type II fucoidan has alternately repeating units of α-(1→3)- and α-(1→4)-linked α-L-fucopyranose (Figure 1) [20,31,33,35]. Several representatives of the orders Chordariales and Laminariales contain Type I fucoidan, whereas those from the order Fucales contain Type II fucoidan [14,19,22,23,36]. The fucose-linked sulfate groups in Type I fucoidan are found in C2- and C4-positions, whereas in Type II fucoidans they are found in C2-, C3- and C4 positions [20,35].

Different sets of fucoidans may be produced by the same species of brown seaweeds. For example, the brown seaweed *Sargassum stenophyllum* synthesizes two different sets of fucoidans [37]. One set of fucoidan contains α-L-fucose as the major component and substantial amounts of other sugars, such as β-D-galactose, β-D-mannose, α-D-glucuronic acid, α-D-glucose and β-D-xylose, with higher percentages of glucuronic acid and fewer sulfate groups located on different sugar units. The other set of fucoidan contains small amounts of α-D-glucuronic acid and high percentages of sulfate groups, which are concentrated on fucose residues, with only fucose and galactose as major components. Similarly, the brown seaweed *Adenocystis utricularis* also produces two different types of fucoidans; one type, called the galactofucan, is mainly composed of L-fucose, D-galactose and ester sulfate groups, whereas the other type, called the uronofucoidan, is composed mainly of fucose along with several other monosaccharides and significant amounts of uronic acids and fewer amounts of sulfate esters [38]. Cui et al. [39] extracted six different kinds of fucoidans from *Saccharina japonica* which varied in their monosaccharide compositions, sulfate contents and molecular weights.

The biological activities of fucoidan, such as the antioxidant and anticoagulant activities, are influenced by the variations in uronic acid and degrees of sulfation [32,40]. The degree of sulfation may vary from 15 to 30% depending on the species of brown macroalgae [35]. The high number of branch points in the macromolecular skeleton increases the complexity of the fucoidan structure [4,19]. 

Fucoidans are generally high molecular weight macromolecular structures [4,40] ranging from approximately 10 kDa [41] to 2000 kDa [42]. The method of extraction is decisive in the molecular size of fucoidan as high temperature induces breaks in the molecule, resulting in fragmentation, whereas the use of strong chemicals can introduce chemical groups into the polysaccharide structure [40].

The fucoidans are anionic owing to the negative charge of the macromolecular skeleton imparted by the presence of sulfate ester groups. The biological interactions of fucoidans with various macromolecular structures, such as proteins, are based on their charge density and fine chemical characteristics [4]. It is a highly hygroscopic polysaccharide and is soluble in water and acidic solutions [31]. The solubility of fucoidan is largely dependent on the level of branching and the number of sulfate groups. Though water-soluble, fucoidans do not yield highly viscous solutions and are not used as gelling agents for industrial applications. However, the addition of NaCl, CaCl_2_ and sugars can increase the dynamic viscoelasticity of fucoidan [43]. The dynamic viscoelasticity of fucoidan in an aqueous solution is determined by several factors, such as the originating species, concentration of fucoidan and its molecular weight, sulfate content, branching points, pH and temperature. The fucoidan obtained from the commercially cultured *C. okamuranus* showed stable dynamic viscoelasticity over a wide range of pH, whereas it decreased with an increase in temperature [43]. Blending with other polymers, such as chitosan [44] and poly(2-hydroxyethyl methacrylate) [45], of opposite net charge can facilitate electrostatic interaction with the negatively charged sulfate groups of fucoidan and thus improve its rheological properties and gel formation. Additionally, blending with such biocompatible polymers makes fucoidan suitable for biomedical applications.

## 5. Extraction, Purification and Structural Modification of Fucoidan

**Figure 2:**
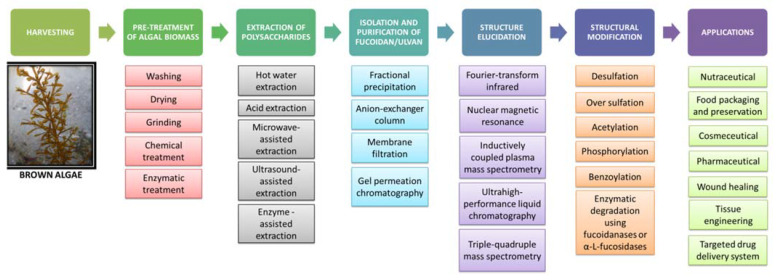
Processes in the extraction, purification and applications of fucoidan.

The sulfated heteropolysaccharides from marine seaweeds are obtained through various time- and resource-intensive sequential steps, such as harvesting, washing, mechanical, chemical or enzymatic pretreatment of algal raw material, followed by extraction of the polysaccharides using various extraction agents, isolation, purification and finally lyophilization and preservation for various intended applications (Figure 2).

### 5.1. Harvesting and Pretreatment of Algal Biomass

Numerous microbes and epiphytes are found inhabiting the surface of macroalgae and they produce polysaccharides which need to be removed by surface sterilization protocol to prevent hampering of the purity of fucoidan. The harvested algae are first washed to remove salt, sand and epiphytes. The washed algae are then dried in shade, and ground into powder to increase the surface area for the action of extraction agents to be used in the subsequent steps and to enhance the release of polysaccharides. The algal biomass contains macromolecules other than fucoidan, such as other polysaccharides, proteins, lipids and pigments, which may blemish the purity of the final product. Chemical or enzymatic pretreatment steps are employed to remove these impurities. Treating the algal biomass with ethanol or acetone removes lipids and pigments [46]. The compounds, such as polyphenyl, chlorophyll and aroma extracts, can be removed by adsorption onto activated charcoal. Chloroform/methanol solvent mixture is also used for the removal of algal pigments [47]. The polysaccharides in algal biomass other than the desired fucoidan can be removed by sequential extraction and precipitation [47]. 

### 5.2. Extraction of Fucoidan

The conventional methods of extraction of fucoidan are hot water extraction and acid extraction [48]. Hot water extraction is performed at 80–90 °C in solutions containing sodium oxalate or ammonium oxalate as divalent cation chelator that can chelate the Ca^2+^ that crosslinks the sulfated polysaccharide strands in the algal cell wall [49]. Acid extraction is performed using dilute HCl [38,49]. The residual algal biomass obtained by filtration or centrifugation after the initial extraction of polysaccharides is usually subjected to several extraction cycles to maximize the yield of polysaccharides. Table 2 summarizes the various extraction methodologies reported for the extraction of fucoidan polysaccharides from marine macroalgal biomass. 

Rodriguez-Jasso et al. [52] employed hydrothermal processes, such as microwave-assisted extraction (MAE) and autohydrolysis (AH), for the extraction of sulfated polysaccharides from *Fucus vesiculosus*. Ultrasound-assisted extraction (UAE) using water/ethanol as the extraction solvent was reported for the extraction of sulfated polysaccharides from the macroalgae *A. nodosum*, *F. vesiculosus* and *Bifurcaria bifurcata* [56] and the Vietnamese brown seaweed *Sargassum mcclurei* [57]. The MAE, AH and UAE extraction methods degrade the algal cell walls and facilitate the release of polysaccharides into the aqueous phase. Hydrothermal-assisted extraction (HAE) in an autoclave apparatus using 0.1 M HCl and further processing by sequential application of ultrasound and thermal technologies were reported for the extraction of polysaccharides from the biomass of brown algae *Laminaria hyperborea* and *A. nodosum* [58]. Innovative technologies, such as HAE, MAE, AH and UAE, are regarded as environmentally friendly and cost-effective techniques as they improve the efficiency of extraction with short extraction times and reduced generation of wastes and use of chemicals [53,58]. Various parameters of extraction, such as temperature, pressure, duration, solvent and sample-to-solvent proportions, influence the yields, physicochemical and biological characteristics, as well as the biological applications of algal polysaccharides [6]. The extraction protocol has a significant impact on the purity, degree of sulfation, molecular weight and monosaccharide composition of the final product.

The extraction and precipitation of sulfated heteropolysaccharides Is a non-specific process and hence the extract and precipitate may contain other polysaccharides, such as starch, cellulose, alginate, etc. Hence, enzymatic or chemical procedures are required for the removal of these impurities and to obtain fucoidan in the purest form possible. Treatment with Na_2_CO_3_ and acetone can precipitate and remove alginate [47]. Enzymatic treatment using cellulase, α-amylase, trypsin, neutral protease and proteinase K can hydrolyze starch, cellulose and proteins from the extract and ensure the purity of the final product [46]. Enzyme-assisted extraction (EAE) is reported as a useful approach for the recovery of industrially important bioactive components, including sulfated polysaccharides, from brown seaweeds [17]. 

Pressurized liquid extraction involving temperature as an important factor is another highly efficient technique for the extraction of fucoidan from algal biomass with a considerably higher extraction yield [51]. The increase in temperature under high pressure disrupts the hydrogen bonding, van der Waals forces and dipole interactions in the cell wall matrix and improves the penetration of extraction liquid into the matrix and diffusion of the polysaccharides into the extraction liquid, thus increasing the extraction yield. 

### 5.3. Isolation and Purification of Fucoidan

The algal extracts obtained through the different extraction protocols are crude mixtures of different polysaccharides, proteins, phenolic compounds and pigments. The crude algal extracts are to be subjected to rigorous purification steps, such as fractional precipitation, ion exchange column chromatography, membrane filtration, size exclusion chromatography or affinity chromatography [59]. The sulfated polysaccharides are less soluble in polar solvents, which facilitate their isolation from the aqueous phase by fractional precipitation using ethanol, calcium chloride [38,47], or using anion exchanger columns [47]. Sulfated polysaccharides are polydisperse and fucoidans of varying molecular weight may be produced by the same algal species. Membrane filtration using different molecular weight cut-off membranes is used for the fractional separation of fucoidans from other polysaccharides and also to separate their different molecular variants [49]. Size exclusion chromatography, also known as gel permeation chromatography, is also used for the fractional separation of fucoidan according to their molecular sizes [21,33]. 

### 5.4. Structure Elucidation of Fucoidan

The composition and structure of sulfated heteropolysaccharides are so highly diverse and complex that the elucidation of the chemical structure of the whole polymer is time- and resource-intensive and requires the use of several techniques. The minutiae of the structural features of polysaccharides can be elucidated by spectrometric techniques, such as Fourier-transform infrared (FTIR), nuclear magnetic resonance (NMR) [33,60,61], inductively coupled plasma mass spectrometry (ICP-MS) [21] and Raman spectroscopy [21]. Ultrahigh-performance liquid chromatography coupled with triple-quadruple mass spectrometry (UHPLC/QqQ-MS) that performs analysis in multiple reaction monitoring (MRM) mode for rapid and simultaneous determination of glycosidic linkages in polysaccharides is also emerging [62]. The combined use of spectrophotometric methods and regio- and stereoselective enzymes can also give insight into their chemical structure [19]. The molecular weight characteristics are determined by gel filtration chromatography [41].

### 5.5. Structural Modification of Fucoidan

Structural modification of sulfated polysaccharides by chemical and enzymatic means can yield novel derivatives of algal polysaccharides with improved and more effective biological activity [63]. The chemical modification involves desulfation, over-sulfation, acetylation, phosphorylation and benzoylation. The acid-based extraction protocol removes the sulfate groups from sulfated algal polysaccharides [5]. The anticoagulant activity of fucoidans from brown algae depends on their molecular weight, the degree of sulfation and the distribution of sulfate groups in the repeating monosaccharide units. The benzoylated derivative of fucoidan extracted from *L. japonica* had strong scavenging activity on superoxide, hydroxyl and 1,1-diphenyl-2-picrylhydrazyl (DPPH) radical [64]. The phosphorylated and aminated derivatives of synthesized fucoidan showed stronger antioxidant ability than native fucoidan [65]. 

Low molecular weight derivatives of algal sulfated polysaccharides with varied biological activities can be obtained by chemical, physical or enzymatic methods. Enzymatic degradation of sulfated algal polysaccharides using fucoidanases or α-L-fucosidases to yield bioactive oligosaccharides is advantageous over chemical methods since the former selectively hydrolyzes the glycosidic bonds, preserving the sulfation pattern essential for biological activity [4,5,66].

## 6. Industrial Production Scenario

From ancient times, seaweeds are traditionally used in Asian countries as herbal medicine for the treatment of several diseases and also as part of a regular diet [67]. Over the past several decades, there has been an escalation in the world seaweed production by aquaculture which surpassed the wild collection. In 2019, the wild collection remained at 1.1 million tons, whereas the cultivation of seaweeds increased to 34.7 million tons and accounted for 97% of the world seaweed production [68]. In 2021, the global commercial seaweed market was USD 15.01 billion, and it is projected to reach USD 24.92 billion in 2028 at a CAGR of 7.51% during the forecast period of 2021 to 2028 [69]. According to the Food and Agriculture Organization of the United Nations, Asia is the world’s largest producer of marine macroalgae, with a contribution of 97.4% to the total production in 2019 [68]. Globally, the leading macroalgae-producing country is China, followed by Indonesia, the Republic of Korea, the Philippines, Japan and Malaysia. In Europe, Spain, France and Ireland have the largest number of macroalgae companies, whereas Norway, France and Ireland produce the largest volumes of seaweed biomass [29]. The American and European countries still depend on the wild collection of seaweeds and the macroalgae aquaculture techniques are to be intensified to increase seaweed production by aquaculture [28,68]. 

The increasing adoption of commercial seaweed as functional foods, cosmeceuticals, nutraceuticals, and pharmaceuticals is expected to propel the seaweed market growth [70]. The commercially cultured brown macroalga *C. okamuranus* is used for the industrial-scale production of fucoidan. The unique rheological properties of this fucoidan have promoted its commercialization in Japan, as an ingredient in drinks and tablets used as a nutritional supplement [43,67]. Fucoidan from *F. vesiculosus* is labelled as GRAS (Generally Recognized as Safe) by the US Food and Drug Administration for use as an ingredient in food [1,71]. The European Union also has approved fucoidan extracts from *F. vesiculosus* and *U. pinnatifida* as novel foods for use in foods and food supplements [24]. Several commercial fucoidans from *F. vesiculosus* and other brown algae species are marketed by well-known companies, such as Sigma-Aldrich^®^, Algues and Mer and Marinova^®^ [4,72]. The commercial fucoidan from *F. vesiculosus* is reported to have great potential to be used as an agent against obesity because of its anti-adipogenic activity [73]. Fucoidan has been applied in different industrial sectors, such as cosmetics, dietary and animal feed supplements [42]. Development of novel fucoidan-based pharmaceutical products for the treatment of cardiovascular diseases is in the pipeline, and its phase I evaluation for human safety is completed [74]. 

## 7. Biological Action and Health Benefits of Fucoidan

Fucoidan is reported to have immense therapeutic potential and health benefits (Table 3). The fucoidans extracted from *L. japonica* have multiple biological actions, including antitumor, antithrombotic, antiatherosclerosis, hypolipidemic, hypoglycemic, antioxidant, anti-inflammatory, renoprotective and immunomodulatory effects [48]. 

### 7.1. Antioxidant Action

Reactive oxygen species (ROS), such as superoxide anion, hydroxyl radical, hydroperoxy radical, lipid peroxide, nitric oxide and peroxynitrite, are produced during aerobic cellular metabolism. The free radical scavenging system that is comprised of various enzymatic and non-enzymatic antioxidants neutralizes the ROS and protects the cell organelles and macromolecular structures, such as DNA, RNA, proteins and membrane lipids, from disintegration. The imbalance between the generation of ROS and the scavenging action of antioxidants makes the cells susceptible to oxidative stress, ultimately leading to various diseases, such as arthritis, neurodegenerative diseases, cancer, etc. Butylated hydroxyanisole (BHA) and butylated hydroxytoluene (BHT) are synthetic antioxidants that are known to have adverse effects on humans [33]. Intense research is directed towards discovering novel non-toxic natural compounds with remarkable antioxidant activity for the prevention of diseases induced by oxidative stress. 

Several reports are available documenting the in vitro and in vivo antioxidant action of fucoidan. The antioxidant activity of fucoidans can be primary [33] or secondary [60]. In primary antioxidant activity, the fucoidans donate electrons to the free radicals, thereby neutralizing them, whereas secondary antioxidants scavenge reactive oxygen by providing electrons to a primary antioxidant and decomposing H_2_O_2_ [40]. Generally, fucoidans have stronger secondary antioxidant activity than the synthetic antioxidants BHA and BHT (Koh et al., 2019). The sulfate content and molecular size of fucoidans are quite variable and this strongly influences their antioxidant action [33,40] akin to the case of other pharmacological actions. The low molecular weight fucoidan extracted from the brown seaweed *U. pinnatifida* harvested in New Zealand has stronger secondary antioxidant activity than BHA [60]. The low molecular weight fraction of fucoidan extracted from the sporophyll of *U. pinnatifida* had significantly higher primary antioxidant activity than the high molecular weight fraction [33]. Both the fractions of *U. pinnatifida* fucoidan had a similar secondary antioxidant activity, which was higher than the antioxidant activity of butylated hydroxyanisole (BHA). Similarly, the fucoidan extracted from Sargassum binderi (F_sar_) has an antioxidant activity that is superior to or similar to the antioxidant action of commercial food-grade fucoidan (F_ysk¬_), whereas the antioxidant potential of both F_sar_ and F_ysk_ is remarkably higher than the synthetic antioxidants BHA and BHT [77]. 

The low molecular weight fucoidan, prepared by radical degradation using gamma irradiation, had stronger 1,1-diphenyl-2-picrylhydrazyl (DPPH) radical scavenging activity than that prepared by acidic hydrolysis [34], indicating that the degradation method employed for the preparation of fucoidan strongly affects the antioxidant activity of fucoidan, in addition to molecular weight. 

The in vitro tests conducted to assess the primary and secondary antioxidant potential of fucoidans include total antioxidant capacity, reducing power, copper chelation, hydroxyl radical scavenging and hydrogen peroxide scavenging. Silva et al. [40] compared the antioxidant activity of three commercial fucoidans from *Macrocystis pyrifera*, *Undaria pinnatifida*, and *Fucus vesiculosus* and demonstrated that the copper-chelating activity of fucoidans is not only dependent on the degree of sulfation but also the distribution of sulfate groups. Among the three fucoidans, the one from *U. pinnatifida* had strong hydroxyl radical scavenging activity. In addition to exhibiting good scavenging action on H_2_O_2_, the three fucoidans also protected the viability of RAW264.7 macrophages and zebrafish *Danio rerio* embryos exposed to H_2_O_2_ with attenuation of the effects of oxidative stress on proliferation rate and other cellular functions [40]. 

Besides being an ROS scavenging antioxidant, fucoidans can also act as preventive antioxidants that block or reduce the generation of ROS and suppress oxidative stress. The low molecular weight fucoidan extracted from the sporophyll of *U. pinnatifida* is capable of inhibiting oxidative stress and mitogen-activated protein kinase activity in the RAW264.7 cell line, thereby suppressing the inflammatory response [85]. All these findings highlight the potential of fucoidans to be commercialized as a functional food ingredient to ameliorate the clinical symptoms of degenerative diseases induced by oxidative stress and improve overall health. 

### 7.2. Anticancer Action/Apoptotic Effect 

Cancer is one of the leading causes of loss of human life. Inducing apoptosis in malignant cells by regulating senescence-associated genes is one of the strategies for the treatment of cancers. Immunomodulation or enhancing the immunity and immune response can potentially restrict the growth of tumors. Hence, immunomodulating compounds can be used as adjuvant chemotherapy without causing any substantial adverse effects on the host. 

The enterocytes that line the Intestinal epithelium interact with various polysaccharide nutrients by endocytosis and antigen uptake and help maintain bowel health [86]. The intestinal epithelial cells may turn malignant when the cell cycle regulation fails and may lead to the development of cancer. The fucoidan extract from *Sargassum cinereum* can curtail the growth of colon cancer cell line Caco-2 in a dose-dependent manner [78]. The treatment of the Caco-2 cell line with fucoidan results in the loss of mitochondrial membrane permeability and increased production of reactive oxygen species (ROS) which induce apoptosis, ultimately leading to the death of cancer cells. Fucoidan exerts differential action on normal cells and cancerous cells [87]. Fucoidan decreased the cell viability by inducing cytotoxicity in CL-6 cholangiocarcinoma cells in a dose-dependent manner, whereas the viability of OUMS normal cells remained unaffected by fucoidan treatment [87]. The increased production of ROS in carcinoma cells treated with fucoidan can be attributed to its ability to cause mitochondrial membrane depolarization [78]. The carcinoma cells show depolarization of mitochondrial membrane on treatment with fucoidan, whereas the normal cells retain their mitochondria in healthy status even after fucoidan treatment [87]. Fucoidan was also shown to induce apoptosis through a caspase-dependent signaling pathway and ROS-mediated mitochondrial pathway in human breast cancer MCF-7 cells [88,89] and human hepatocellular carcinoma SMMC-7721 cells [90]. In hepatocellular carcinoma SMMC-7721 cells [90] and human breast cancer MDA-MB-231 and MCF-7 cells [89], the increase in the levels of ROS in fucoidan-induced apoptosis was also associated with decreased consumption of glutathione. Zhang et al. [89] demonstrated oxidative stress induced by fucoidan in combination with other chemotherapeutic agents as an important event in the death of cancer cells.

Fucoidan can modulate multiple signaling pathways, thereby suppressing cancer cell survival, tumorigenesis and metastasis. Chantree et al. [87] demonstrated the apoptotic action of fucoidan in cholangiocarcinoma CL-6 cells. In cholangiocarcinoma CL-6 cells fucoidan induces the up-regulation of apoptotic marker proteins, such as Bax, which then alters the mitochondrial membrane potential, resulting in the translocation of cytochrome c from mitochondria into the cytosol and further activation of signaling pathways for caspase cascades. Furthermore, in carcinoma cells, fucoidan causes the down-regulation of anti-apoptotic marker proteins, such as Bcl-2. The carcinoma cells show extensive nuclear chromatin condensation and DNA fragmentation on treatment with fucoidan. Fucoidan can arrest the cell cycle of carcinoma cells in G_0_/G_1_ phase by suppressing the PI3K/Akt signaling pathway, resulting in the down-regulation of Cyclin D and CDK4 [87,88].

Chen et al. [91] have demonstrated that fucoidan modulates endoplasmic reticulum stress cascades and induces apoptosis in cancer cells via different mechanisms in a cell type-dependent manner. In metastatic MDA-MB-231 breast cancer cells, fucoidan induces apoptosis by down-regulation of glucose-related protein 78 (GRP78). The down-regulation of GRP78 leads to ER Ca^2+^ leak into the cytosol which in turn activates CaMKII phosphorylation and Bcl-associated X protein (Bax) and caspase 12 expression inducing apoptosis. In the metastatic colon cancer cells HCT116, fucoidan induces cell death by down-regulating the expression of the survival protein ERp29. Fucoidan was also shown to exert an inhibitory effect on the ER-stress-related cell survival cascade IRE-1\XBP-1s in both MDA-MB-231 breast cancer cells and HCT116 colon cancer cells. Moreover, fucoidan-induced ER stress also promotes cell death via phosphorylation of elongation factor 2α (elF2α) and up-regulation of ATF4\CHOP pro-apoptotic cascade [91]. Ma et al. [92] established that in hepatocellular carcinoma MHCC-97H cells, fucoidan activates the up-regulation of the tumor suppressor gene LINC00261 which inhibits the cell proliferation and invasion by scrounging the miR-522-3p and increasing the expression level of SFRP2. Fucoidan induced apoptosis in MHCC-97H cells in both in vitro and in vivo studies. 

Presently, only a few clinical trials are conducted to establish the anticancer action of fucoidan despite its beneficial potential. Fucoidan modulates the immune response and improves the prognosis of cancer survivors. In an open-label clinical study conducted for advanced cancer patients, Takahashi et al. [93] evaluated the efficacy of fucoidan focusing on its anti-inflammatory action in improving their quality of life. The advanced cancer patients with metastases orally administered with fucoidan demonstrated a significant reduction of main pro-inflammatory cytokines, such as interleukin-1β (IL-1β), IL-6 and tumor necrosis factor-α (TNF-α) after two weeks of fucoidan ingestion. In another clinical study conducted on cancer survivors orally administered with fucoidan, Nagamine et al. [94] demonstrated that ingestion of fucoidan extracted from Cladosiphon okamuranus significantly enhanced the activation of natural killer cells in male cancer survivors. The anticancer action and apoptotic efficacy of fucoidan suggest its potential to be developed as a promising therapeutic regimen against various cancer cell types [78]. 

### 7.3. Immunomodulating Action

Sulfated polysaccharides exert the immunomodulatory action by acting as either a promoter or an inhibitor of the immune response [95]. The sulfated polysaccharides extracted from the brown seaweed *Sargassum hemiphyllum* showed inhibition of lipopolysaccharide-induced inflammatory response in the mouse macrophage cell line (RAW264.7) by a significant reduction in the secretion profiles of pro-inflammatory cytokines, including interleukins (IL-1β, IL-6), tumor necrosis factor-α (TNF-α) and nitric oxide (NO) [80]. 

Low molecular weight polysaccharides extracted from marine macroalgae have remarkably higher immunomodulatory action than their high molecular counterparts since the increase in molecular weight potentially limits their application owing to a corresponding decrease in the solubility, absorptivity and bioavailability of these polysaccharides. The fucoidans extracted from two brown seaweed species, *Sargassum crassifolium* and *Padina australis*, show intestinal immunomodulating activity via Peyer’s patch cells [79]. 

The heterogeneity of fucoidan plays a pivotal role in modulating the immune response. By and large, fucoidans with low sulfate content and high heterogeneity have high immunomodulatory action. Fucoidans modulate the immune response by interacting with the scavenger receptors present on the surface of macrophages [39]. Cui et al. [39] demonstrated the immunomodulatory action of six kinds of fucoidans extracted from the brown seaweed *Saccharina japonica* on the mouse macrophage cell line (RAW264.7). The heterogeneity of six fucoidans facilitated their interaction with structurally heterogenous scavenger receptors on the surface of macrophages, thereby modulating the immune response. The different fucoidans from *S. japonica* exert immunomodulatory action by up-regulating the expression of IL-6, IL-1β and TNF-α at the protein level [39]. The fucoidan is also reported to induce nitric oxide production via p38 mitogen-activated protein kinase and NF-κB-dependent signaling pathways that lead to the activation of iNOS promoter, expression of iNOS mRNA and iNOS protein in RAW264.7 macrophages [39,96]. 

### 7.4. Lipolytic and Anti-Adipogenic/Anti-Obesogenic Activity

The fucoidan extracted at a commercial scale from the brown seaweed *F. vesiculosus* is reported to have anti-adipogenic and lipolytic activity [73]. Oliveira et al. [73] extracted four different fractions of fucoidan (F0.5/F0.9/F1.1/F2.0) from *F. vesiculosus*, all of which had lipolytic action on the adipocytes. Furthermore, the F1.1 and F2.0 fractions demonstrated an anti-adipogenic effect, preventing the differentiation of pre-adipocytes by inhibiting the expression of key adipogenic proteins (C/EBPα, C/EBPβ and PPARγ). On the contrary, the other two fractions, F0.5 and F0.9, had an adipogenic effect. The F2.0 fraction with anti-adipogenic action and three times higher lipolytic action than the other fractions can be used as an anti-obesogenic agent. 

### 7.5. Hepatoprotective Action 

**Figure 3:**
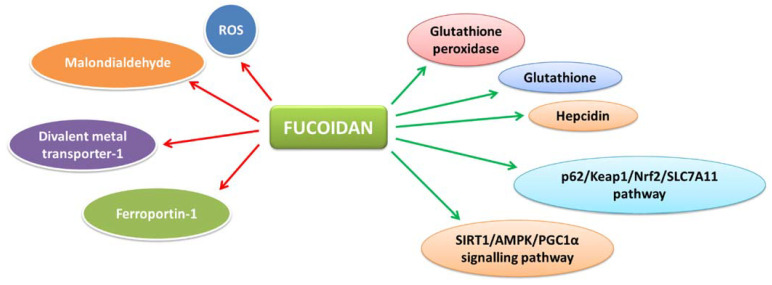
Hepatoprotective action of fucoidan. Fucoidan increases the levels of glutathione peroxidase-4, glutathione and hepcidin; ameliorates alcohol-induced ferroptosis damage in the liver by up-regulation of p62/Keap1/Nrf2/SLC7A11 pathway; activates SIRT1/AMPK/PGC1α signaling pathway in non-alcoholic liver disease. (Green arrows indicate increase in synthesis or up-regulation of gene expression). Glutathione peroxidase and glutathione scavenge free radicals. Hepcidin reduces iron absorption through ubiquitin-dependent proteasome degradation of divalent metal transporter-1 (DMT1); p62 recruits ubiquitinated Keap1 proteins to autophagosomes and promotes expression of Nrf2; Nrf2 promotes downstream gene transcription of SLC7A11; SLC7A11 is a transmembrane protein responsible for the cystine/glutamate antiporter to import cystine for glutathione biosynthesis and antioxidant defense. Fucoidan activates SIRT1/AMPK/PGC1α signaling pathway which reduces lipotoxicity-related oxidative stress and inflammation. Fucoidan reduces ROS in hepatocytes; reduces the levels of malondialdehyde, divalent metal transporter-1 and ferroportin-1 to prevent iron overload. (Red arrows indicate decrease in synthesis or activity).

The accumulation of reactive oxygen species (ROS) and iron in hepatocytes lead to ferroptosis, an iron-dependent form of programmed cell death characterized by depletion of glutathione, inactivation of glutathione peroxidase-4 and increased levels of lipid peroxides [97]. Chronic alcohol consumption and administration of several drugs can lead to oxidative stress and iron overload in hepatocytes, which can cause steatosis or fatty liver in the early stages and progressively lead to liver fibrosis or cirrhosis and ultimately even end up in liver cancer. Fucoidan is reported to exert hepatoprotective action (Figure 3) in alcoholic [98] and non-alcoholic fatty liver disease [99] by inhibiting oxidative stress and inflammation without affecting the normal liver cells. Intragastric administration of fucoidan in rats with ferroptosis-induced liver injury on exposure to alcohol was shown to reduce the levels of ROS and malondialdehyde in hepatocytes while increasing the levels of antioxidants, such as glutathione peroxidase and glutathione [98]. Moreover, fucoidan alleviated liver damage due to increased deposition of iron and formation of ferritin. In unison, fucoidan increased the expression of hepcidin and decreased divalent metal transporter-1 and ferroportin-1. Fucoidan exerts the reversal of ferroptosis-induced liver damage by the up-regulation of p62, Nrf2, SLC7A11 and GPX4, having cytoprotective roles against lipid peroxidation injury [98]. In non-alcoholic fatty liver disease, the low molecular weight fucoidan from L. japonica was shown to ameliorate lipotoxicity-related oxidative stress and inflammation by activation of the SIRT1/AMPK/PGC1α signaling pathway [99]. 

### 7.6. Neuroprotective Action 

Fucoidan has the remarkable potential to protect the survival of nerve cells and ameliorating learning and memory impairment in neurodegenerative disorders, such as dementia and Alzheimer’s disease [100] and motor impairment in Parkinson’s disease [101]. Fucoidan exerts a protective role in nerve cells by reducing oxidative stress, enhancing the mitochondrial respiratory function [101], inhibiting the caspase-dependent apoptosis pathway and regulating the cholinergic system [100,102]. The neuroprotective action of fucoidan derived from *U. pinnatifida* sporophylls was demonstrated in PC12 cells derived from a pheochromocytoma of rat adrenal medulla and damaged by infusion with β-amyloid and D-galactose [100]. Fucoidan protects the nerve cells from the harmful impact of oxidative stress by preventing the release of cytochrome *c* from the mitochondria to cytosol, inhibiting the expression of caspase-3 [102,103], increasing the expression of apoptosis inhibitor proteins (IAPs), such as livin and X-linked IAP, improving the antioxidant activity by activation of superoxide dismutase and glutathione and reduction of malondialdehyde [103], and thus ultimately regulating apoptosis [100]. Regulation of the cholinergic system of nerve cells by fucoidan was demonstrated in Alzheimer’s disease model mice as an increase in the activity of acetylcholine and choline acetyltransferase and a decrease in the activity of acetylcholine esterase [100]. In the monocrotophos-induced Alzheimer’s disease model of *Drosophila melanogaster*, fucoidan was found to increase the brain expression of neurotransmitters, such as dopamine, glutamate, GABA, octopamine, serotonin and tryptamine, thus highlighting the effect of fucoidan in maintaining neurotransmission [102].

The immunohistochemistry and immunoblotting studies conducted in MPTP-treated Parkinsonic mice using fucoidan extracted from *Turbinaria decurrens* confirmed its neuroprotective effect on the dopaminergic neurons, with an increase in the levels of antioxidants, dopamine, dopamine transporter and tyrosine hydroxylase [104]. MPTP (1-methyl-4-phenyl-1,2,3,6-tetrahydropyridine) is a neurotoxin that destroys the dopaminergic neurons in the substantia nigra and corpus striatum of the brain and causes Parkinson’s disease. Fucoidan extracted from *L. japonica* could successfully alleviate dopaminergic neuron degeneration and motor impairment in a rotenone-induced rat model of Parkinson’s disease by enhancing the mitochondrial respiratory function of dopaminergic neurons through the PGC1α/Nrf2 pathway [101].

Fucoidan derived from *L. japonica* could ameliorate the cognitive deficits in the adenine-induced chronic kidney disease (CKD) mice model by inhibition of oxidative stress via the GSK3-Nrf2-HO-1 signaling pathway [105]. The cognitive deficits were improved in adenine-induced CKD mice when orally administered with fucoidan which manifested as an improvement in recognition memory and spatial memory. In addition, fucoidan improved the inflammatory response by inhibiting M1 microglial/macrophage polarization in the hippocampus and promoting M2 microglia/macrophage polarization in the kidney and attenuating cognitive behavior-related hallmark gene expression in the CKD mice model. Similarly, the hippocampal amyloid β-infused rat model of Alzheimer’s disease showed an improved memory function when fed with fermented low molecular weight fucoidan [106]. Fucoidan improved memory impairment by modulating glucose metabolism and gut microbiota in rats induced with Alzheimer’s disease symptoms. The modulation of glucose metabolism is exerted by potentiation of hippocampal insulin signaling via enhancing the pSTAT3→pAkt→pGSK-3β pathway and rectifying the defect of cerebral glucose metabolism by increasing serum concentrations of acetate and butyrate. In addition, fucoidan also increased the expression of ciliary neurotrophic factor and brain-derived neurotrophic factor in the hippocampus [106]. 

Fucoidan is effective in the reversal of neurotoxicity and depressive behavior induced by alcohol consumption. Fucoidan treatment increases 5-hydroxytryptamine and brain-derived neurotrophic factor levels in serum and brain tissues, which alleviates the depressive behavior in alcohol withdrawal mice [107]. The alcohol-induced high levels of TNF-α and IL-1β can be reverted and IL-10 and TGF-β levels can be increased by fucoidan treatment. Fucoidan down-regulates the TLR4/MyD88/NF-κB p65 pathway, increases the expression of CD68 in the hippocampus and inhibits the activation of microglia cells which are the chief cells involved in the inflammatory response related to neurotoxicity induced by alcohol consumption. Fucoidan administration alleviates the depression-like behaviors induced by alcohol consumption through the modulation of the gut–microbiota–brain axis [107].

### 7.7. Anticoagulant Action 

Coagulation is an inherent mechanism of hemostasis to prevent blood loss in injuries. Heparin, a highly sulfated polysaccharide belonging to the glycosaminoglycan family in mammalian tissues has long been used as an anticoagulant, but it has side effects, such as excessive hemorrhage, heparin-induced thrombocytopenia, etc. [108]. Fucoidan from marine macroalgae has been recognized as an efficient anticoagulant alternative. The fucoidan isolated from the brown seaweed *Turbinaria decurrens* possess anticoagulation properties, and the silver nanoparticles (AgNPs) synthesized using the extracted fucoidan showed increased anticoagulant activity compared to the fucoidan polymer [47]. 

### 7.8. Antibacterial Action 

Marine macroalgae find applications in the pharmacological sector owing to their antimicrobial potential against drug-resistant pathogens [109]. The macroalgal compounds may have intrinsic antimicrobial properties or they can potentiate the effect of other antimicrobial compounds via inhibition of efflux pumps [110]. Similar to other biological actions, the antimicrobial activity of seaweed polysaccharides also depends on molecular weight, charge density, sulfated content and structural and conformation characteristics [111]. The antimicrobial polysaccharides from macroalgae interact with the glyco-receptors of the bacterial cell wall and membranes, leading to the disruption of membrane stability and cellular functions and protein leakage [112]. 

The sulfated polysaccharides from several brown algae, such as *L. japonica*, *Laminaria digitata*, *A. nodosum*, *Sargassum muticum* and *U. pinnatifida*, have antibacterial action against pathogenic bacteria, such as *Listeria monocytogenes*, *Staphylococcus aureus*, *Escherichia coli*, *Salmonella enteritidis* and *Pseudomonas aeruginosa* [111,113]. Seaweed extracts from brown algae with antimicrobial action against fish pathogens are also used as prophylactic and/or therapeutic agents in feed for aquaculture [111]. 

Low molecular weight and strong anionic properties enhance the antibacterial property of fucoidans, as depolymerized fucoidans have more excellent antibacterial activity than unprocessed fucoidans. The depolymerized fucoidan from *L. japonica* effectively inhibited the growth of *Escherichia coli* and *Staphylococcus aureus*, whereas the native fucoidan had no antibacterial activity [76]. The depolymerized fucoidan destroys the cytomembranes by targeting membrane proteins, resulting in altered membrane fluidity and/or activated autophagocytosis.

Fucoidans can enhance the pharmacological action of other drug compounds in battling against pathogenic bacteria by exerting a synergistic effect [112]. Lee et al. [114] evaluated the antibacterial activities of fucoidans extracted from several species of brown algae against cariogenic and periodonto-pathogenic bacterial strains when used in combination with ampicillin and gentamicin. The antibiotics showed a reduction of bacteria growth to a greater degree when used in synergism with fucoidans than when used alone. 

### 7.9. Antiviral Action 

The marine sulfated polysaccharides of macroalgal origin are reported to exert antiviral activity against a broad spectrum of viruses by intervening in different stages of a viral infection, such as virus attachment, penetration, un-coating of the virus capsid inside the host cell, and transcription and translation of viral genomes [115]. The sulfated polysaccharides from marine macroalgae find application in the development of antiviral drugs for the prevention of communicable diseases in humans and animals. 

The fucoidan extracted from *F. vesiculosus* enhances the host’s innate immune response and inhibits the replication of human norovirus (hNoV), which causes acute gastroenteritis. The antiviral action of fucoidan demonstrated on hNoV-infected zebrafish larvae is exerted through the up-regulation of the expression of a series of interferon-stimulated genes that encode antiviral effectors, including helz2, marco and rsad2 (viperin) [84].

The fucoidan from the sporophyll of *U. pinnatifida* exerts antiviral activity against the influenza virus by interfering with the transcription of the viral genome [83]. The bioactive fucoidans from *F. vesiculosus* [116] and *Sargassum swartzii* [82] have been reported to inhibit the reverse transcriptase activity of human immunodeficiency virus-1. The fucoidans extracted from the three brown seaweeds, *Sargassum mcclurei*, *S. polycystum* and *Turbinara ornata*, inhibit HIV-1 infection by blocking the early steps in the entry of HIV into the target cells [117]. 

The two purified fucoidans (SHAP-1 and SHAP-2) from *Sargassum henslowianum* are active against herpes simplex virus-2 and the antiviral mechanism is through blocking the HSV-2 virion adsorption to host cells [81]. The native and enzymatically modified fucoidans from *Fucus evanescens* were shown to exert antiviral activities against herpes simplex viruses (HSV-1 and HSV-2), enterovirus (ECHO-1), and human immunodeficiency virus (HIV-1) in Vero and MT-4 cell lines [18]. The antiviral action of *Fucus evanescens* fucoidan was more prominent against HSV-2 and significant inhibition of virus-induced cytopathic effect (CPE) was reported. The intraperitoneal administration of native and enzyme-modified fucoidan was effective in protecting the mice model from lethal intravaginal HSV-2 infection [18]. 

The fucoidan from *C. okamuranus* is reported to prevent infection by Newcastle Disease Virus (NDV). NDV belongs to the paramyxoviridae family, which includes pathogens causing morbidity and mortality worldwide in humans and animals [49], especially in birds and resulting in drastic losses in the worldwide poultry industry [15]. The mixture of fucoidan and another sulfated polysaccharide ulvan inhibits cell–cell fusion in NDV infection, indicating that they are promising antivirals in combating paramyxovirus infections [49]. The fucoidan from *C. okamuranus* prevents the penetration of NDV into the host cell and the antiviral activity exhibited was higher than the Ribavirin antiviral control [15]. 

### 7.10. Cosmeceutical Applications

According to Murphy and Dow [118], the macroalgae extracts can have one of the three main functions in cosmeceutical formulations: (1) as additives that improve product stabilization, preservation, and/or organoleptic properties; (2) as excipients that constitute the transport medium for bioactive ingredients; and (3) as true functional compounds with cosmeceutical effects.

Fucoidans have commendable cosmeceutical applications. Skin ageing and wrinkle formation are caused by the action of skin matrix enzymes, such as collagenase and elastase. The degraded collagen fibrils accumulated in the extracellular matrix of skin prevent the proliferation and differentiation of new skin tissues and induce further enzyme activity, leading to skin degradation in a positive feedback loop. Inhibiting the activity of skin matrix enzymes can prevent skin ageing by reducing skin degradation and may also induce the formation of a new skin matrix [119]. The polysaccharides from macroalgae are reported to have skin bioactive properties under in vitro and in vivo conditions. The fucoidan extracts from *U. pinnatifida* and *F. vesiculosus* can inhibit collagenase and elastase enzymes related to skin ageing [42]. Both extracts can up-regulate the SIRT1 protein, which enhances the catalysis of sugars and lipids and causes the skin to appear more youthful. The fucoidans from *U. pinnatifida* and *F. vesiculosus* are also efficient in soothing the skin, reducing the depth of wrinkles and in giving protection from UV rays. The *F. vesiculosus* extract has a high polyphenol content which confers additional efficacy in antioxidant and skin-brightening applications [42]. 

The purified fucoidan extracts of *F. vesiculosus* as active components in the creams and lotions for skin applications can provide anti-ageing and anti-wrinkle benefits [120]. Fucoidan is reported as an anti-inflammatory ingredient in cosmetics, allergic-condition-soothing products or post-surgical formulations [121]. Furthermore, fucoidan can perform skin protection functions by increasing the association of matrix metalloproteinase (MMPs) with their specific tissue inhibitors of matrix metalloproteinases (TIMPs). In ex vivo experiments, fucoidan was shown to diminish human leukocyte elastase activity, thereby protecting the human skin elastic fiber network from enzymatic proteolysis by serine proteinase [75]. Fucoidan can down-regulate melanin synthesis and thereby assuage skin pigmentation and can be used as an ingredient in skin-whitening formulations [119]. Fucoidan is accepted as an ingredient for skin conditioning [24].

## 8. Biomedical Applications of Fucoidan

**Figure 4:**
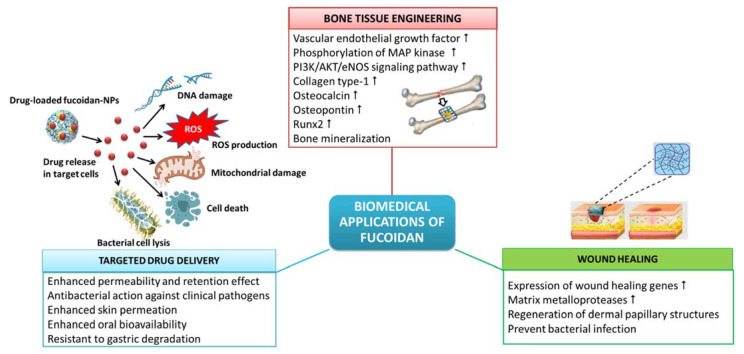
Biomedical applications of fucoidan.

The beneficial properties of fucoidan, such as biocompatibility, to cell attachment and proliferation, biodegradability, eco-friendliness, etc., have made fucoidan a sustainable and promising alternative to synthetic polymers in the preparation of biomaterials for different biomedical applications, comprising drug delivery, tissue engineering, wound healing, etc. (Figure 4). 

### 8.1. Wound Healing

Biomaterials from natural polysaccharides are widely recommended for the treatment of dermal wounds and wound dressing applications since they are non-toxic and highly biocompatible [44]. Hydrogel films are suitable for wound dressing applications as they can provide a moist environment for the wound and absorb the wound exudate, thus accelerating wound healing [122]. The hydrogels should have antibacterial action to prevent infection of the wound and facilitate wound healing. 

The *U. pinnatifida* fucoidan can enhance and modulate the wound healing response of the skin by increasing the expression of wound healing genes for the matrix metalloproteases [42]. The fucoidan/chitosan hydrogels prepared using the fucoidan from *F. vesiculosus* were demonstrated to have high efficiency in the treatment of dermal burns with the best regeneration of dermal papillary structures and fastest closure of wounds when tested in adult male rabbits [44]. 

### 8.2. Tissue Engineering and Regenerative Medicine 

Algal polysaccharide-based hydrogels find extensive biomedical applications in tissue engineering and regenerative medicine as repair matrices with pre-seeded cells and bioactive growth factors. Tissue repair and regeneration, e.g., bone repair, requires the proliferation of cells, establishment of cell–cell communication and formation of new blood vessels or angiogenesis. Kim et al. [123] elucidated the mechanism behind the fucoidan-induced osteoblastic differentiation in human mesenchymal stem cells and angiogenesis. Fucoidan-induced osteoblast differentiation is concomitant with a remarkable increase in the synthesis of mRNA and a corresponding increase in the secretory expression of vascular endothelial growth factor leading to angiogenesis. Additionally, the conditioned media obtained from the fucoidan-induced mesenchymal cells also demonstrated angiogenic activities, such as increased phosphorylation of mitogen-activated protein kinase and PI3K/AKT/eNOS signaling pathway. Kim et al. [123] also demonstrated the therapeutic potential of fucoidan in osteoblast differentiation and angiogenesis in vivo in a rabbit model with calvarial bone defects, wherein fucoidan hastened the formation of new blood vessels and promoted bone formation partially. Akin to this, the fucoidan from *Sargassum ilicifolium* also has the potential for bone tissue engineering since it can induce alkaline phosphatase activity, bone mineralization and expression of osteoblast-specific genes in mesenchymal stem cells [55]. 

Hybrid biomaterials made of combinations of ceramics, hydroxyapatite and other polymers, such as fucoidan, collagen, chitin and chitosan, are more biocompatible and biodegradable. Such hybrid biomaterials can mimic the extracellular matrix and the mechanical properties of bone and are considered to provide more promising results in bone repair than individual materials [124]. In an in vivo biomimetic process, Ahn et al. [124] evaluated the bioactivity of hydroxyapatite-fucoidan nanocomposites which revealed no toxic effect on adipose-derived stem cells. Moreover, hydroxyapatite-fucoidan nanocomposites also significantly increased the expression of early and terminal stage osteogenic differentiation markers, such as collagen type-1, osteocalcin, osteopontin and runx2, and induced mineralization for bone formation. Moreover, hydroxyapatite-fucoidan nanocomposites induced bone formation in rabbit with a defective tibia, suggesting it to be a compatible biomaterial for bone tissue engineering [124]. 

### 8.3. Targeted Drug Delivery Systems

Drug delivery systems using nanoparticles (NPs) envisage targeted therapy which is aimed at executing the pathophysiological mechanism termed enhanced permeability and retention (EPR) effect [125]. EPR is a pathophysiological mechanism employed in cancer therapy, in which the drugs conjugated with nano-sized polymeric drug carriers can progressively accumulate in the tumor vasculature and deliver and retain relatively higher drug concentrations in targeted solid tumors [126]. This improves the efficacy of anticancer compounds while reducing their side effects on non-targeted cells. Moreover, the NP carriers also improve the chemotherapeutic efficacy of the entrapped drug by modulating their pharmacokinetics and bio-distribution profile [125]. Pawar et al. [125] developed an efficient, safe and immunocompetent nanoparticle platform for the targeted delivery of doxorubicin against breast cancer by electrostatically assembling fucoidan with cationic polyethylenimine. The cytotoxicity, cell cycle arrest and apoptotic effect on tumor cells were highly enhanced in doxorubicin conjugated with fucoidan NPs in comparison to the free drug. Furthermore, the pharmacokinetics of doxorubicin entrapped in fucoidan NPs revealed preferential drug localization in tumors [125] indicating its excellent and safe use as an immunomodulating chemotherapeutic against cancer. 

The anionic fucoidan can physically crosslink with cationic groups of polymers, such as collagen, gelatin, alginate and chitosan, through electrostatic interactions forming hydrogels [127,128]. The hydrogels made from fucoidan can be loaded with bioactive compounds and used as exceptional drug delivery carriers because of their porous nature, which facilitates the controlled release of drugs. Moreover, the adhesiveness of polysaccharides can increase the interaction of drugs with the cells and prolong their action, bringing about the desired pharmacological action. Fucoidan-based drug delivery systems can be made with their dimensions in the nano-scale, forming nanogels [128]. The fucoidan extracted from *Turbinaria decurrens* and the fucoidan-coated AgNPs showed excellent antibacterial activity against clinical pathogens, with a more pronounced action against the Gram-negative bacteria (*Pseudomonas aeruginosa* MTCC 2642 and *Escherichia coli* MTCC 40) over the Gram-positive bacteria (*Streptococcus mutans* MTCC 896 and Staphylococcus aureus MTCC 96) [47]. The antibacterial action of fucoidan-coated AgNPs was more prominent than the fucoidan polymer since the NPs provide a higher surface-to-volume ratio that facilitates increased interaction with the bacterial cell surface. The close interaction of fucoidan-coated AgNPs with the bacterial cell enhances the dissociation of fucoidan by the enzymatic action of the bacterial cell and releases silver ions into the bacterial cell [47]. The adhered nanoparticles can increase membrane permeability and lead to the disruption of the bacterial cell wall [129]. The hydrogel biofilms made of the biocompatible polymer poly(2-hydroxyethyl methacrylate) embedded with fucoidan were reported to have good antibacterial activity in ophthalmic application [45]. Fucoidan also finds applications in the development of NPs and hydrogels for the topical delivery of drugs in the treatment of skin-related inflammatory diseases. In contrast to the conventional topical anti-inflammatory applications, drugs encapsulated in fucoidan NPs or hydrogels present enhanced retention time and distribution of topical drugs. Barbosa et al. [130] developed fucoidan/chitosan NPs for the enhanced skin permeation of the anti-inflammatory drug methotrexate. The methotrexate-loaded fucoidan/chitosan NPs were biocompatible with fibroblasts and keratinocytes and significantly inhibited pro-inflammatory cytokines, such as IL 1-β, IL-6 and tumor necrosis factor-α. 

The presence of acidic and basic functional groups In the polymeric structure confers pH-sensitivity to fucoidan that can elicit a response to changes in external pH and makes it applicable in pH-responsive drug delivery systems. Coutinho et al. [127] developed mucoadhesive and pH-responsive fucoidan/chitosan NPs for the oral delivery of methotrexate in lung cancer therapy. The pH-responsive fucoidan/chitosan NPs loaded with methotrexate were resistant to challenges, such as pH and gastric degradation. The mucoadhesive property of fucoidan/chitosan NPs enhances the oral bioavailability of the drug. Methotrexate-loaded fucoidan/chitosan NPs are biocompatible to fibroblasts while mediating apoptosis in lung cancer cells [127]. In another study, self-assembled zein-fucoidan complex NPs were developed as a delivery system for the controlled release of resveratrol, a health-promoting dietary polyphenol, and were demonstrated to have low cytotoxicity to normal human intestinal epithelial cell line-6 cells [131]. 

## 9. Food and Feed Applications of Fucoidan 

About 85% of the global macroalgal production comprises food products for human consumption [24]. Until now, only two macroalgal products are authorized by the European Union as novel foods for use in food and food supplements: the fucoidan extracts from *F. vesiculosus* and *U. pinnatifida*; and the phlorotannins extracted from the brown alga E. cava [24]. However, fucoidans are also found in edible species, such as *L. japonica* and *C. okamuranus* [132]. 

Fucoidan has a positive impact on the growth performance and productivity of animals. The effect of fucoidan on animal health is through modulating the gut environment for the growth of beneficial gut microflora, stimulating the innate immune system and reducing the risk of diarrhea [132]. The commercially marketed algal products TascoTM from *A. nodosum* and Ocean FeedTM (a blend of brown, green and red macroalgae) are used as animal feed additives to improve the performance and immune response [132,133].

## 10. Food Packaging and Preservation

Biodegradable polymers, being environmentally friendly, are attractive alternatives to plastics to be used as food packaging materials. In addition to eco-friendliness, chemical safety is of paramount importance when developing food contact materials, to prevent the migration of hazardous chemicals to foods from the packaging materials [134]. Macroalgae containing 25–60% of the dry biomass weight accounted for by polysaccharides are gaining increased interest as bioplastics for food packaging materials. In Germany, macroalgae are developed into edible carton-like packaging materials [24]. 

**Figure 5:**
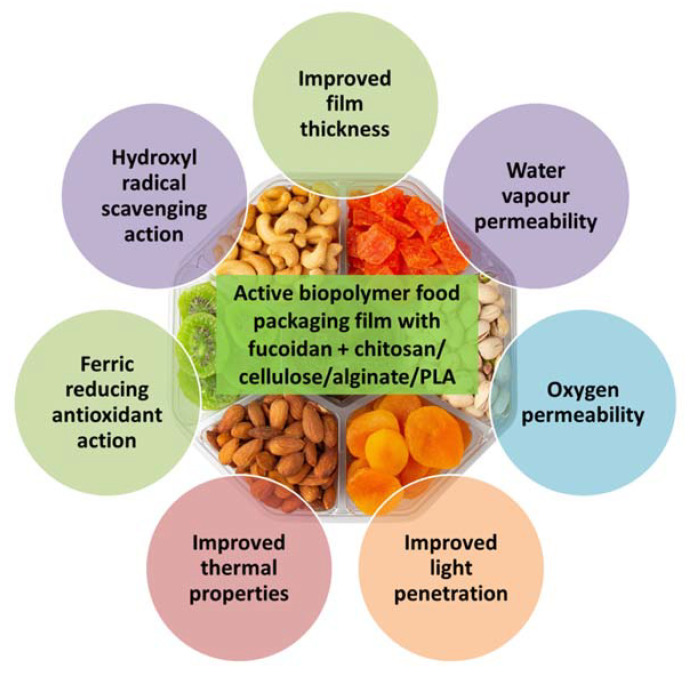
Beneficial properties of active biopolymer food packaging films prepared from fucoidan blended with other polymers, such as chitosan, cellulose, alginate or polylactic acid.

Active packaging films are an innovative approach to food packaging and preservation that is gaining interest due to their optical, structural, thermal, antioxidant and antimicrobial properties [135,136]. According to European regulation (EC) No 450/2009, active materials and articles mean materials and articles that are intended to extend the shelf-life or to maintain or improve the condition of packaged food; they are designed to deliberately incorporate components that would release or absorb substances into or from the packaged food or the environment surrounding the food. Two types of active packaging systems are in practice: active scavenging systems or absorbers that remove undesirable substances, such as moisture, carbon dioxide, oxygen, ethylene or odor molecules from the packed food or its surroundings; and active releasing systems or emitters that release favorable compounds, such as antimicrobial compounds, antioxidants, flavor, ethylene or carbon dioxide, to the packed food or headspace to extend shelf life and enhance the safety and sensory characteristics without loss of product quality [137]. Doh et al. [138] manufactured seaweed nanocomposite biopolymer films reinforced with cellulose nanocrystals using the brown seaweeds kombu (*L. japonica*) and sargassum (*Sargassum natans*). The seaweed biopolymer film had improved physical properties (Figure 5), such as thickness, moisture content and water solubility. The barrier properties to water, oxygen and light were reinforced in the biopolymer films and the thermal properties were also enhanced. The antioxidant properties of the seaweed biopolymer film were also improved, with the kombu film having higher antioxidant properties than the sargassum film. An activated biodegradable polylactic acid (PLA) film containing 8% lyophilised alga *Fucus spiralis* and 1% sorbic acid was developed for the preservation of fish megrim *Lepidorhombus whiffiagonis* under refrigeration [139]. The samples wrapped in activated PLA films maintained sensory quality and were still acceptable on day 11, whereas the fish specimens wrapped in polyethylene films were not acceptable by that time. 

Edible packaging films made of edible ingredients are increasing in demand because of their safety of consumption and ease of use [136]. Edible films with natural antioxidant properties were developed by Gomaa et al. [140] using alginate and fucoidan extracted from the brown macroalga *Sargassum latifolium* and chitosan derived from the fungus *Aspergillus niger*. The water vapor permeability, oxygen permeability and film thickness were increased by the incorporation of fucoidan and/or Ca^2+^ into the alginate-chitosan edible films, which also showed good barrier properties against ultraviolet light. Fucoidan blending also decreased the water solubility of the film and improved the film moisture content at equilibrium. The alginate-chitosan-fucoidan blended edible films also showed good antioxidant properties with ferric reducing antioxidant power and hydroxyl radical scavenging activity. 

## 11. Challenges and Outlook

With the growing applications of seaweed polysaccharides, there is an increasing demand for seaweed biomass which cannot be met alone by harvesting from the wild. Despite the few reports on nutrient sources for land-based cultivation [24,26,27,141], the search for a suitable nutrient source that can support large-scale production of marine macroalgae is still a major bottleneck to be overcome. Hence, aquaculture practices are to be intensified and robust management practices are to be envisaged for the maximum production of seaweed biomass and bioactive polysaccharides. The co-extracted compounds diminish the purity of fucoidan and affect the reproducibility of results in clinical trials [46,47]. Hence, it is very pertinent to formulate homogenous production, extraction and purification strategies for the utmost purity and quality of the final product that can ensure reproducible results in physicochemical characterization and clinical trials. 

Several factors, such as the source of synthesis, molecular weight, degree of sulfation, presence of co-extracts, the composition of sugars, degree of branching, etc., influence the biological actions of fucoidans. Chemical or enzymatic modification of the polysaccharides would increase their biological actions and applicability in nutraceutical, pharmaceutical and cosmeceutical sectors. Hence, the successful application of these sulfated polysaccharides for the nutritional and health benefits of humans and animals requires the characterization of these influencing parameters. 

The application of fucoidan in food contact packaging materials requires a sustainable and economic resource of high-quality fucoidan with bioactive properties that can prolong the shelf life of food without any waning in quality or nutritional status. Moreover, developing edible food packaging materials requires that the fucoidan is free from any toxic materials originating from the microbes that may inhabit the macroalgal surface. Hence, arduous protocols are to be strategized for the isolation and purification of fucoidan to ensure quality and purity.

Enthrallingly, the anticancer action of fucoidan relies on its potential to inhibit tumorigenesis and progression through any of the mechanisms, such as regulation of tumor immunity, inhibition of angiogenesis, halt of the cell cycle and/or induction of apoptosis [92]. The anticancer action of fucoidan varies with different cell types of carcinoma [91]. The signaling pathways underpinning the cellular regulatory mechanisms and the role fucoidan can deliver in these pathways remain largely elusive and deciphering these mechanisms have broad projections in the field of future cancer therapy. Despite the potential benefits of fucoidans in combating cancer, only a few clinical trials are evaluating the efficacy of fucoidans. Moreover, extensive studies are much awaited to establish the safety levels of doses and effective serum levels of fucoidans for the inhibition of tumor growth or metastasis. The pharmacokinetic interaction and synergistic action of fucoidan with other chemotherapeutic agents and its role in alleviating the side effects of chemotherapy would be much applauded for improving the prognosis and quality of life of cancer survivors [93]. Larger controlled preclinical and clinical trials are ardently needed to evaluate and establish the pharmacological actions of fucoidan in targeted and combinatorial chemotherapy in the treatment of different forms of carcinoma. Though dietary supplementation of fucoidan is suggested [94], the bowel absorption and serum availability of fucoidan need to be investigated. Furthermore, fucoidan is found to exert differential action in normal cells and damaged or cancerous cells. In the cancerous cells fucoidan induces the production of ROS, causing oxidative stress leading to apoptosis, whereas in hepatocytes and nerve cells damaged by chronic alcohol consumption [99,107] and in nerve cells showing neurodegenerative symptoms [100,101,102], fucoidan exerts protective action by reducing oxidative stress and preventing apoptosis. The viability of normal cells remains unaffected by fucoidan treatment [87,99]. Hence, it is very pertinent to decipher the molecular mechanism behind how fucoidan distinguishes between normal and damaged or malignant cells to exert its differential action, which would give a new direction in the development of targeted chemotherapeutics.

The global prevalence of dementia is on the rise owing to population ageing and population growth [142] and it is important to further explore the neuroprotective action of fucoidan in modulating the potential metabolic risk factors and regulating the signal pathways associated with dementia and other neurodegenerative disorders. 

Fucoidan has remarkable potential as a biomaterial for tissue engineering and wound healing applications owing to its biodegradable nature and proliferative action. However, the exact signaling mechanisms involved in the cell adhesion, proliferation and differentiation and the influence of fucoidan in these mechanisms are less well understood and demand a further detailed investigation. Extensive in vivo studies in animal models are required to corroborate the biological actions of fucoidan in tissue engineering applications. In addition, clinical evidence on safety evaluation is still limited for fucoidan-incorporated nanoparticles in drug delivery applications. 

## 12. Conclusions

Fucoidan is an exceptional and propitious natural bioactive polysaccharide derived from marine macroalgae. The biomedical, pharmaceutical and food industry target fucoidans, owing to their biological actions that benefit human health. For any biomedical or pharmaceutical application or food-contact packaging application, onerous purification protocols are to be employed to provide fucoidan of high purity. Though the in vitro and in vivo studies reported hitherto elucidate the biological action of fucoidan that warrant a comparable efficacy in therapeutic applications, currently there is a significant paucity of clinical trials to corroborate the safety of these products for human use. Moreover, the comprehensive molecular mechanism behind the various biological actions of fucoidan remains largely recondite and calls for intensified research interventions to bridge the knowledge lacunae. By demystifying the mechanism of biological action and with the advancement of successful clinical trials, fucoidan from marine macroalgae can emerge as commercial products contributing to the blue bioeconomy.

## Figures and Tables

**Table 1:** Marine macroalgal sources of fucoidan polysaccharides.

Marine Macroalga	Chemical Composition/Structure	Reference
*Fucus evanescens*	([→3)-α-L-Fuc*p*(2,4O SO_3_^−^)-(1→4)-α-L-Fuc*p*(2OSO3−)-(1→])_n_	[18]
*Sargassum horneri*	repeating →3-α-l-Fuc*p*(2 SO_3_^−^)-1→4-α-l-Fuc*p*(2,3SO(3)(-))-1→ fragment, with insertions of →3-α-l-Fuc*p*(2,4SO(3)(-))-1→ fragment	[19]
*Laminaria longipes*	[→3)-α-l-Fuc*p*-(2SO(3)-)-(1→4)-α-l-Fuc*p*-(1→2)-α-l-Fuc*p*-(4 SO_3_^−^)-(1→]_n_	[20]
*Laminaria hyperborea*	(1→3)-α-L-fuco-pyranose (31.9%) to be the dominant residue, followed by 1→2-linked (13.2%) and 1→4-linked (7.7%) fuco-pyranose	[21]
*Fucus evanescens*	([→3)-α-L-Fuc*p*(2,4O SO_3_^−^)-(1→4)-α-L-Fuc*p*(2OSO_3_-)-(1→]_n_)	[18]
*Ascophyllum nodosum*	[→3)-α-l-Fuc(2SO_3_−)-(1→4)-α-l-Fuc(2,3diSO_3_−)-(1]_n_	[14]
*Fucus evanescens*	[→3)-α-l-Fuc*p*(2SO_3_−)-(1→4)-α-l-Fuc*p*(2SO_3_−)-(1→]_n_	[22]
*Fucus distichus*	[→3)-α-l-Fuc*p*-(2,4-di-SO_3_−)-(1→4)-α-l-Fuc*p*-(2SO_3_−)-(1→]_n_	[23]

**Table 2:** Methods of extraction of fucoidan from macroalgal biomass.

Macroalgal Species	Extraction Method	Extraction Yield/Efficiency	Reference
*Ascophyllum nodosum*	Microwave-assisted extraction	16.08%	[50]
*Fucus vesiculosus*	Pressurized liquid extraction at high temperature	25.99 ± 2.22%	[51]
*Fucus vesiculosus*	Microwave-assisted extraction	18.2 ± 1.4%	[52]
*Fucus vesiculosus*	Autohydrolysis process	16.5 ± 1.2%	[52]
*Fucus vesiculosus*	Microwave-assisted extraction	18.22%	[53]
*Nizamuddinia zanardinii*	Ultrasound-assisted extraction	3.51%	[54]
*Sargassum myriocystum*	Enzyme-assisted extraction	6.2%	[46]
*Turbinaria decurrens*	Soaking in chloroform/methanol, sequential extraction in CaCl_2_, HCl	5.58% (crude) 1.28% (purified)	[47]
*Sargassum ilicifolium*	Probe sonication–microwave assisted extraction method Hot water extraction method	8 ± 0.9% 6 ± 0.5%	[55]

**Table 3:** Biological actions and applications of fucoidans from brown macroalgae.

Macroalgal Source	Biological Action	Mechanism of Action	Application	Reference
*Ascophyllum nodosum*	Dermatological action	Inhibition of gelatinase A secretion and stromelysin 1 induction by interleukin-1β on dermal fibroblasts Increasing the association of MMPs with their specific inhibitors, namely TIMPs Minimize human leukocyte elastase activity Protection of human skin elastic fiber network against proteolysis by serine proteinase	Treating inflammatory pathologies with uncontrolled extracellular matrix degradation	[75]
*Cladosiphon okamuranus*	Antiviral action	Inhibition of viral entry into host cell, formation of syncytia and plaque forming units by blocking F protein	Antiviral to prevent New Castle Disease Virus infection in poultry	[15]
*Fucus evanescens*	Antiviral action	Preventive effect, virucidal effect and inhibition of virus adsorption and early stages of virus replication	Broad spectrum antiviral against DNA and RNA viruses, such as herpes simplex viruses (HSV-1, HSV-2), enterovirus (ECHO-1), and human immunodeficiency virus (HIV-1)	[18]
*Fucus vesiculosus*	Anti-adipogenic action	Decrease the expression of key proteins of adipogenic differentiation (C/EBPα, C/EBPβ, and PPARγ)	Treatment of obesity	[73]
*Fucus vesiculosus*	Antioxidant action Dermatological action	Inhibition of skin aging by increasing the expression of *SIRTI*. Improve skin immunity, soothing and protection, age spot reduction	Topical application for skin brightening	[42]
*Laminaria hyperborea*	Anticoagulant action	Inhibition of coagulation proteins Inhibition of complement activation by monocytes Inhibition of platelets	Potential alternative to heparin	[21]
*Laminaria japonica*	Antibacterial	Bactericidal action through destruction of cytomembranes targeting the membrane proteins, which can result in changed membrane fluidity and/or activated autophagocytosis.	Potential for partly or totally replacing antibiotics against *Escherichia coli* and *Staphylococcus aureus*	[76]
*Laminaria longipes*	Anticancer action	Prevent growth of cancer cells Sensitization of cancer cells to X-ray radiation	Effective against melanoma and colon cancer cells	[20]
*Saccharina cichorioides*	Anticancer action	Prevent growth of cancer cells Sensitization of cancer cells to X-ray radiation	Effective against melanoma and colon cancer cells	[20]
*Sargassum binderi*	Antioxidant	Free-radical scavenging activity (DPPH), reducing power, superoxide anion scavenging activity (SOA) and hydroxyl radical scavenging activity (OH)	Attenuation of inflammatory cytokines, such as IL-1β, IL-1 and TNF-α, and the degradation of phosphorylated p38 MAPK, ERK1/2 and JNK. Inhibition of iNOS and COX-2 expression induced by lipopolysaccharides	[77]
*Sargassum cinereum*	Anticancer action	Dose-dependent inhibition of growth of colon cancer cells (Caco-2) by induction of apoptosis, increase in ROS production and augmentation of mitochondrial membrane permeability	Promising therapeutic regimen against various cancer cell types	[78]
*Sargassum crassifolium* and *Padina australis*	Immunomodulation	Intestinal immunomodulating activity via Peyer’s patch cells	Maintenance of bowel health	[79]
*Sargassum duplicatum*	Anticancer action	Prevent growth of cancer cells	Effective against colon cancer	[61]
*Sargassum hemiphyllum*	Anti-inflammatory effect	Reduction of secretion profiles of pro-inflammatory cytokines, including IL-1β, IL-6, TNF-α, and NO Dose-dependent inhibition of lipopolysaccharide-induced mRNA expressions of IL-β, iNOS, and COX-2 Down-regulation of NF-κB	Treatment of inflammation	[80]
*Sargassum henslowianum*	Antiviral action	Reduction of plaque forming units Block virion adsorption to host cells	Treatment of Human Simplex Virus (HSV-1 and HSV-2) infection	[81]
*Sargassum myriocystum*	Antioxidant action	Free radical scavenging activity against hydroxyl and DPPH radical	Treatment for various oxidative stress and age-related diseases	[46]
*Sargassum swartzii*	Antiviral action	Reduction in HIV-1 p24 antigen levels and reverse transcriptase activity	Potential as an anti-HIV-1 agent	[82]
*Undaria pinnatifida*	Antioxidant	Secondary antioxidant capacity	Can replace synthetic antioxidant butylated hydroxyanisole (BHA) in treatment of diseases related to oxidative stress	[60]
*Undaria pinnatifida*	Antiviral action and immunomodulation	Inhibit replication of influenza A virus Stimulate both innate and adaptive immune defense functions in virus-infected host	Development of new therapeutic options, including its combination with neuraminidase inhibitors, such as oseltamivir	[83]
*Fucus vesiculosus*	Antiviral action	Inhibit viral replication Enhance host innate immune response through up-regulation of interferons signaling related genes and interferon-stimulated genes encoding antiviral effectors	Effective against human noroviruses (hNoV)	[84]

## Data Availability

The data presented in this study are available in the literature cited.
